# The effect of decongestion on nasal airway patency and airflow

**DOI:** 10.1038/s41598-021-93769-6

**Published:** 2021-07-13

**Authors:** Qiwei Xiao, Alister J. Bates, Raul Cetto, Denis J. Doorly

**Affiliations:** 1 Center for Pulmonary Imaging Research, Cincinnati Children’s Hospital Medical Center, Cincinnati, OH USA; 2 Division of Pulmonary Medicine, Cincinnati Children’s Hospital Medical Center, Cincinnati, OH USA; 3 Department of Pediatrics, University of Cincinnati, Cincinnati, OH USA; 4 Department of Aeronautics, Imperial College London, South Kensington Campus, London, SW7 1AZ UK

**Keywords:** Physiology, Engineering, Computational science

## Abstract

Nasal decongestant reduces blood flow to the nasal turbinates, reducing tissue volume and increasing nasal airway patency. This study maps the changes in nasal anatomy and measures how these changes affect nasal resistance, flow partitioning between superior and inferior cavity, flow patterns and wall shear stress. High-resolution MRI was applied to capture nasal anatomy in 10 healthy subjects before and after application of a topical decongestant. Computational fluid dynamics simulated nasal airflow at steady inspiratory flow rates of 15 L.min$^{-1}$ and 30 L.min$^{-1}$. The results show decongestion mainly increases the cross-sectional area in the turbinate region and SAVR is reduced (median approximately 40$\%$ reduction) in middle and lower parts of the cavity. Decongestion reduces nasal resistance by 50$\%$ on average, while in the posterior cavity, nasal resistance decreases by a median factor of approximately 3 after decongestion. We also find decongestant regularises nasal airflow and alters the partitioning of flow, significantly decreasing flow through the superior portions of the nasal cavity. By comparing nasal anatomies and airflow in their normal state with that when pharmacologically decongested, this study provides data for a broad range of anatomy and airflow conditions, which may help characterize the extent of nasal variability.

## Introduction

The human nose acts as the initial conduit for inhaled air, while simultaneously conditioning, filtering, and sensing the inhaled volume. Many pathologies, such as allergic rhinitis, rhinosinusitis, nonallergic rhinitis, nasal polyps, and the common cold can cause nasal obstruction^1^, restricting airflow and affecting the secondary functions of the nose. Therefore, there is a significant interest in characterizing nasal airflow in healthy subjects and those with disease. The goal of these studies is to aid clinical decision making aimed at treating nasal pathologies. Nasal airflow is difficult to measure *in vivo*, so many studies have used computational fluid dynamics (CFD)^2–4^ or experimental methods such as particle-image velocimetry (PIV)^5–8^ to model respiratory airflow. It is observed that CFD under-predicts resistances determined by rhinomanometry^9^. Certainly the fact that nasal resistances are determined at higher transnasal pressure losses than common in restful breathing makes direct comparison difficult, since geometries would need to be acquired at a matching, sustained and relatively forceful inhalation. However previous studies have shown CFD and rhinomanometry are well-correlated, so although both methods have limitations, CFD can provide good insight^9,10^.

Nasal obstruction can be treated surgically^11^. Several studies have investigated the application of CFD to improve surgical success rates^12–16^. These studies focused on the effect of local modifications to nasal structure aimed at reducing nasal obstruction over a limited number of subjects. The findings from these studies demonstrate how nasal cavity anatomical structures affect nasal resistance. In addition, Suhyla et al.^17^ evaluated the effect of turbinectomy on the secondary nasal function olfaction using CFD. Nasal airflow simulations have also been used to review the effectiveness of surgical interventions^18^. We are not however aware of previous studies which have sought to relate the differing changes in anterior vs posterior nasal cavity on their corresponding contributions to overall nasal resistance.

Temporary alleviation of nasal obstruction is also commonly effected via the application of nasal decongestant^19^, especially for treatment of acute rhinosinusitis^20^. Nasal decongestant reduces bloodflow to the erectile tissue within the nose, reducing the volume of the nasal turbinates and increasing the patency of the nasal passages. Larissa et al. have shown decongestant can significantly reduce nasal airway resistance^21^ through *in vivo* measurements, however the effect of decongestion on nasal anatomy and nasal airflow, including both the overall anatomical and localised anatomical changes, have not been well quantified, especially over a sizeable cohort. Therefore, the first aim of this paper is to quantify the changes in nasal airflow due to the application of nasal decongestant to provide a reference for clinicians when comparing the reduction in nasal resistance that may be achieved via nasal decongestant.

Nasal anatomy might also vary with time due to a process known as the nasal cycle: the periodic expansion and contraction of erectile tissue within one side of the nose, followed by the other side. Heetderks et al. found that 80$\%$ of 60 subjects exhibited this nasal cycle^22^ and similarly, Hasegawa et al. found 72$\%$ of 50 subjects had a clearly defined nasal cycle using rhinomanometry^23^. Each side of the cavity congests and decongests repeatedly over a period between 25 min to 8 h^24^, and the cycle has been observed to continue for at least 7 days^25^. Eccles et al. found that unilateral nasal cavity resistance changed almost fourfold due to the nasal cycle^26^. However, most of the subject specific CFD or experimental studies are based on nasal geometries obtained from medical imaging which captures the airway at a single time-point^27–29^. The second aim of this study is to use nasal decongestant to explore the range over which nasal anatomy and airflow parameters can vary in order to map the extremes of nasal geometry and nasal resistance. This information serves as an indication of the maximum range of nasal function that could occur throughout the day, rather than just at the instant that is captured by medical imaging and to provide data on the range of variation for nasal anatomical and aerodynamic parameters that may be applied to future studies based on single time-point imaging.

## Materials and methods

### Subjects

There is a large degree of inter-subject variability in nasal anatomy^30^, which Zhao et al.^31^ demonstrated across 22 healthy subjects. However most CFD studies are limited in the number of subjects they consider by the computational expense of performing CFD simulations^32^.

This study used 10 volunteers aged between 21 and 38. All subjects had no nasal complaints and no rhinoscopic abnormalities on anterior rhinoscopy and fibreoptic nasoendoscopy and subjects with a score less than 7 of sino-nasal outcome test (SNOT-22) were chosen. The SNOT-22 is a validated 22-item CRS-specific QoL instrument which is scored using a Likert scale and has been widely used to assess symptom severity and quality of life for chronic rhinosinusitis^33,34^. Furthermore, subjects with nasal disease or other clinically significant diseases were also excluded.

Ethical approval was granted by the National Health Service (NHS) Health Research Authority NRES Committee South East Coast-Surrey with reference number 06/Q0602/18. Informed consent was obtained from each of the subjects included in this study. All methods were carried out in accordance with relevant guidelines and regulations.

### Imaging and procedures

Anatomical MRI scans were obtained on a 3T GE Healthcare Discovery MR750. Images were acquired in the coronal plane with a spatial resolution of [0.35, 0.35, 1.2] mm. Images were acquired at two conditions: before and 10 min after the application of topical decongestant.

Subjects remained immobilized between scans with their heads placed in a coil and 3 drops of topical alpha adrenergic agent xylometazoline HCL 0.1$\%$ drops were administered by an experienced Ear, Nose and Throat (ENT) surgeon. Topical nasal decongestants are fast-acting, potent drugs which are especially effective for the reduction of nasal congestion. They can be classified into two major groups: (1) sympathomimetic amines (cocaine, amphethamine, adrenaline, ephedrine) and (2) imidazolines (oxymetazoline and xylometazoline). Of the imidazolines, oxymetazoline, a selective − 1 and partial − 2 agonist, and xylometazoline, an − 2 agonist, are the most popular and clinically used derivatives. Both achieve their decongestive effect via activation of-adrenergic receptors, resulting in vasoconstriction of the blood vessels and, consequently, resumption of nasal airflow. Onset of action occurs within 5–10 min for a duration of action of 5–6 h^35^.

### Creation of virtual nasal model

The MR images were segmented, and a 3D surface mesh was created by an experienced ENT surgeon using software package Mimics 15.0 (Materialise, Leuven, Belgium, https://www.materialise.com).

**Figure 1:**
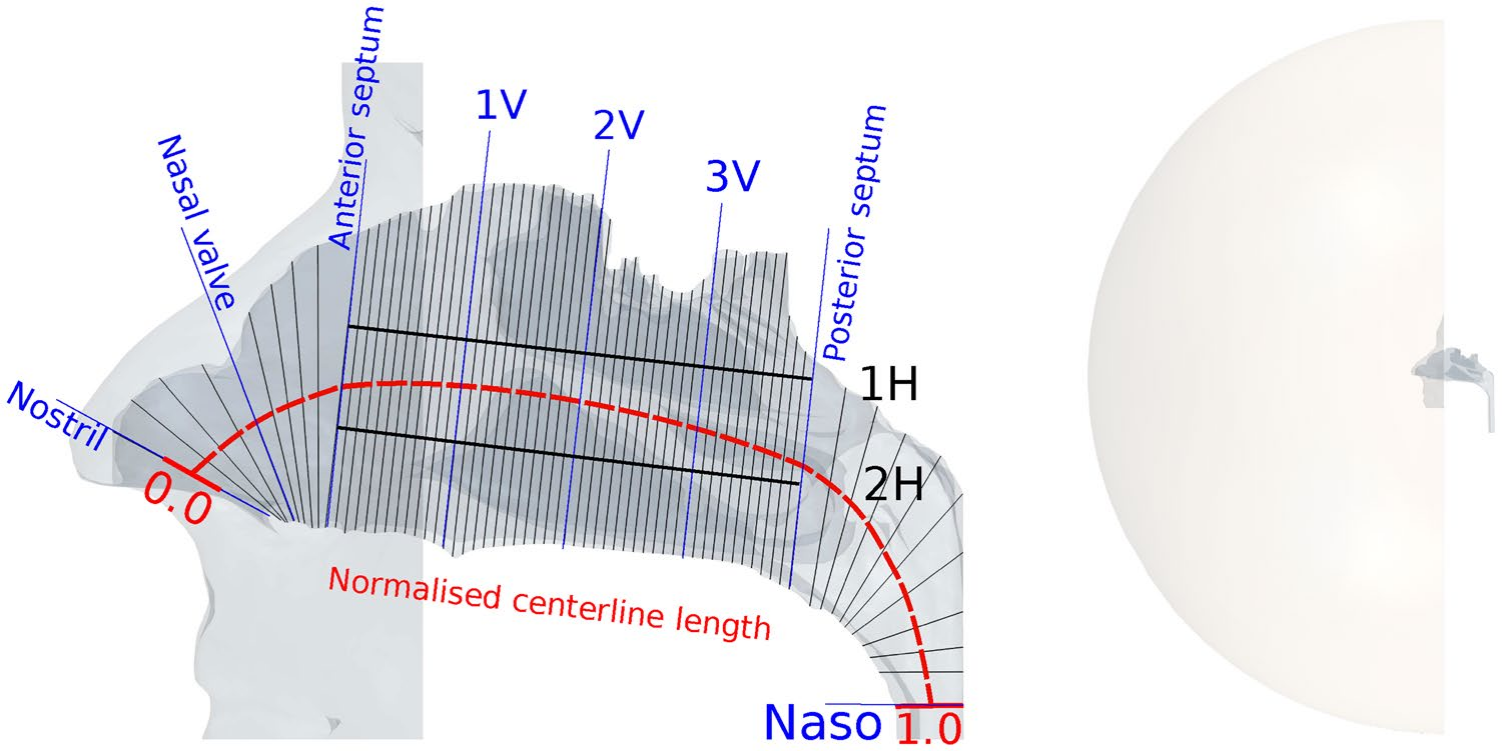
Left side of the figure shows 3D surface view of one normal nasal cavity (Subject-D). Blue lines represent planes defined according to nasal cavity anatomical landmarks. The red dashed line represents the nasal cavity centerline labeled with normalized distance from 0 to 1. Grey lines represent coronal cross-sectional planes normal to the centerline. 1H and 2H black lines divide nasal cavity into three partitions vertically. Right side is the view of the boundary setup in CFD solver, and the half sphere attached with nasal surface ensures a natural inflow profile.

Figure 1 shows a 3D nasal geometry in grey. In order to provide an anatomical framework to compare similar locations in different subjects, the geometry is labelled with anatomical landmarks (black and blue lines). These landmarks represent features such as the nostril, nasal valve, septum and nasopharynx. Vertical planes 1V, 2V, and 3V equally divide the nasal airway between anterior and posterior septum. Horizontal planes 1H and 2H divide the nasal cavity into inferior, middle and superior sections. These lines equally divide the nose between the hard palate bone and a line extending from posterior wall of the sphenoid sinus to the nasal bony septum. The red line indicates the geometric centerline of the nasal airway; measuring from anterior to posterior, the centerline length is normalized to a value of 0 at the nostril and 1 at the nasopharynx, which is defined above the tip of the epiglottis. This facilitates the comparison of nasal anatomies among different subjects.

The anatomical geometry created from MRI extends from the anterior nose to nasopharynx, as shown in Fig. 1. However, the domain of the CFD simulation was extended at the inlet and outlet. A hemisphere of diameter 0.5 m was attached to the exterior face to act as a flow inlet. Doorly et al.^36^ showed the velocity of air approaching the nose during inhalation reduces rapidly. An extension of this size is more than sufficient to ensure a natural flow profile develops at the entrance to the nose; we have not investigated the minimum diameter needed. Previous study have shown that a smaller extension of order of 50 mm diameter is sufficient for modelling inhalation^36^. At the outlet in the nasopharynx, an extruded tube was generated to prevent reverse flow at the outlet.

### CFD simulation methodology and boundary conditions

The commercial software STAR-CCM+ (https://www.plm.automation.siemens.com) was used to solve for the flow. This CFD package solves the governing Navier–Stokes equations using the finite volume method. In this formulation, transport equation of a general property of flow termed as $\phi$ can written in discretized form as follows:

1 $$\begin{aligned} \frac{d}{dt}(\rho \phi V) + \sum [\rho \phi (\vec {u}\cdot \vec {a})]_{f} = \sum (D\nabla \phi \cdot \vec {a})_{f} \end{aligned}$$

where *t* (s) is time, *f* represents the face of each element surface; *V* ($m^3$) is the volume; *D* ($m^2$/s) is the diffusion coefficient; $\vec {a}$ represents a surface cell and $\rho$ ($kg/m^3$) is flow density.

Each simulation consisted of approximately 4 million polyhedral elements with 7 prism layers at the near-wall region. Quasi-steady transient simulations were performed (i.e. transient simulations with constant boundary conditions) with a time step of 1 ms. For each subject, two mass flow rates were simulated: 15 L.min$^{-1}$ represents the mean flow rate during the inspiratory phase of restful breathing of an adult, while 30 L.min$^{-1}$ represents the peak flow rate during the inspiratory phase^37–39^. A zero velocity gradient was applied at the outlet (also known as pressure outlet). All other surfaces were treated as a rigid wall with the non-slip condition (zero velocity at the surface). Nasal airflow was assumed to be incompressible and laminar due to the low velocity of nasal airflow^15,36,40,41^. Li et al.^37^ showed that laminar studies achieve suitable results for the 15 L.min$^{-1}$ flowrate. Zhao et al.^42^ showed for flowrates up to 55 L.min$^{-1}$ there was no obvious difference in flow results between laminar model and k-$\omega$ turbulent models. In addition, we also conducted k-$\omega$ and large eddy simulations, and the total pressure loss between those simulations showed minor differences (of order 0.4$\%$) as tabulated in Appendix E. The effects of gravity and buoyancy were neglected.

Both spatial and temporal convergence studies were conducted. The mesh independence study was conducted, ranging from 2.5 million to 6.3 million (for both normal and decongested states in a representative geometry) and for time steps ranging from 5 ms to 0.1 ms. The difference in trans-nasal total pressure loss was at most 1.2$\%$ between that obtained for a mesh containing approximately 4 million elements, run with a 1 ms time step, versus the corresponding loss measured using 6.3 million mesh elements run with a time step of 0.1 ms. The unsteady simulations revealed a small amplitude flapping motion occurs in the nasopharynx, which is typically where transitional disturbances are most amplified. This results in an oscillation of the total pressure loss of order 1$\%$, which would account for the failure of steady laminar flow solvers to converge, but this is not significant for flow distribution within the cavity or for pressure loss. Appendix A shows the mesh view on multiple pre-defined cross-sectional planes within the nasal cavity.

### Geometric analysis of the nasal cavity

The cavity cross-sectional area (CSA) and the perimeter of each cross-sectional plane were calculated before and after decongestion to analyze the changes in local geometry. The cavity volume (CV) and surface area (SA) of each nasal cavity was measured bounded by the planes at the nostril and nasopharynx and the ratio between them was calculated to produce the surface area to volume ratio (SAVR). All the geometrical measures are presented in a bilateral manner which is a summation of the left and right side of the cavity.

### Nasal airflow measurements

Bilateral results for a flow-related quantity $\phi$ were calculated as a massflow weighted summation between left and right cavity. The massflow weighted summation is defined by the equation below:

2 $$\begin{aligned} \phi _{bilateral} = \frac{\dot{m}_{left}\cdot \phi _{left} + \dot{m}_{right}\cdot \phi _{right}}{\dot{m}_{left} + \dot{m}_{right}} \end{aligned}$$

where $\phi$ represents a general quantity.

#### Reynolds number

Reynolds number is defined as

3 $$\begin{aligned} Re = \frac{\rho U d}{\mu } \end{aligned}$$

where $\rho$ (kg/m$^3$) is the fluid density, *U* (m/s) is mean flow velocity, *d* (m) is the length scale, and $\mu$ (Pa.s) is fluid dynamic viscosity.

The nasal cavity cross-sectional plane is not a circle, so the hydraulic diameter ($d_h$), defined in Eq. (4) is used as a representative length scale in the calculation of Reynolds number.

4 $$\begin{aligned} d_h = \frac{4 A}{P} \end{aligned}$$

In Eq. (4), *A* ($m^2$) and *P* (*m*) denote respectively area and perimeter of the airway cross section, respectively. The mean velocity in Eq. (3) is equal to the pipe flow rate divided by the cross-sectional area, $U=\frac{Q}{A}$. Substituting this relation and the hydraulic diameter (Eq. 4) into the Reynolds number equation (Eq. 3), and cancelling the area terms gives

5 $$\begin{aligned} Re = \frac{4 \rho Q}{\mu P} \end{aligned}$$

where *Q* ($m^3/s$) is flow rate. Since air can be considered incompressible with constant viscosity in respiration, Reynolds number at a given flow rate is therefore inversely proportional to the perimeter of the cavity cross-section.

#### Pressure and resistance

Resistance is defined as the ratio between pressure and flowrate through the cross-sectional planes. In this study, the mean total pressure on each cross-sectional plane was used to calculate resistance.

On each one of the series of cross-sectional planes, the surface-averaged total pressure is calculated then divided by the flow through the plane. The incremental difference in mean pressure between planes divided by the volumetric flow rate gives the contribution of the intraplane portion to the total resistance; cumulative resistance is simply the addition of resistance up to the point of interest.

#### Data analysis

Boxplots are used to illustrate the results, where upper and lower edges of the box indicate the first and third quartile points respectively, outliers are marked with a red cross and a horizontal red line indicates the median. The maximum and minimum value are represented by top and bottom bars. To test the significance of changes observed, the Wilcoxon signed-rank test was used^43^. The null hypothesis for the paired data set is that the median differences have a distribution centred about zero. Following convention, a 95$\%$ confidence level is chosen as the threshold to distinguish significant from non-significant results^44,45^, to indicate whether random variation produced the observed results with the caveat that p-value are not by themselves enough to gauge actual significance^46^.

## Results

### Normalised nasal anatomy

Table 1 shows the mean location and corresponding standard deviation of each anatomical landmark as a fraction of distance along cavity centerline. The bottom row shows the mean distance of each landmark from the nostril plane in millimeters. A full table of results for each subject is in Appendix B.

**Table 1:** Mean location of anatomical landmark (defined by planes) of all the subjects in both normalised units and millimeters. The third row lists the normalised standard deviation of each plane location. *Nos* Nostril plane, *NV* Nasal valve plane, *AS* Anterior septum, *PS* Posterior septum, *Naso* Nasopharynx plane. The anatomical landmarks of each subject are in similar locations with respect to the normalized cavity length, with the maximum standard deviation less than 5$\%.$

Plane	Nos	NV	AS	1V	2V	3V	PS	Naso
Mean	0	0.12	0.18	0.32	0.45	0.58	0.72	1
STD	0	0.03	0.04	0.04	0.03	0.04	0.04	0
Mean(mm)	0	13.5	20.3	36.0	50.6	65.3	81.0	113

### Effect of decongestant on nasal anatomy

#### Cross-sectional area

**Figure 2:**
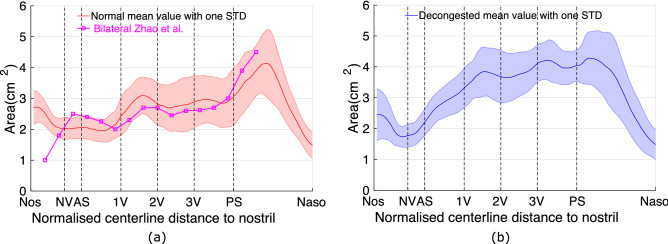
Comparison of mean cross-sectional area between normal (pre-decongested) and decongested cavities, e.g (**a**,**b**). The horizontal axis is the normalised centerline distance, labelled with anatomical markers defined in Fig. 1. The shaded area indicates the variations within the range of one standard deviation. The magenta line (in sub-figure (**a**)) is the same measurement from Zhao et al.^31^ which shows good agreement with the current data. Decongestion increases the cavity cross-sectional area in most parts of the nasal cavity, with the exception around nasal valve area (plane NV).

Figure 2 shows the intranasal CSA for both normal (red) and decongested (blue) cavities. The horizontal axis starts from the location of nostril plane and ends at the nasopharynx plane (as described in Fig. 1). The landmarks defined previously (Table 1) are also indicated as vertical dashed lines labelled along the horizontal axis.

For normal state nasal cavities, CSA is approximately constant between plane AS and 1V. In contrast, for decongested cavities the CSA area rapidly expands between these planes, from 2.2 cm$^2$ at plane AS to 3.3 cm$^2$ at plane 1V. From plane 1V to PS, CSA is relatively constant along each nasal cavity but is significantly larger in the decongested cases (mean 3.8 cm$^2$ vs 2.8 cm$^2$). This represents an increase in CSA of 34.8$\%$ due to decongestant before the posterior septum.

Changes in the anterior nose can highly affect how inhaled air is directed in the nasal cavity. Figure 2 suggests the CSA of the anterior nose in the region of the nasal valve reduces slightly, but this is likely not significant, see below. The CSA clearly increased predominantly in the posterior aspect of the nasal cavity as that is the region surrounded by the most erectile tissue.

Figure 2a also shows the mean CSA measured along the nasal cavity measured by Zhao et al. from a cohort of 22 healthy adults. Zhao et al’s study measured CSA normal to the anterior-posterior axis rather than normal to a centerline. Their results show good agreement to these nasal cavity measurements throughout the turbinate region. However their results deviate in the region of the nostril, since the airway centerline used here as reference direction rotates approximately 90 degrees from the anterior-posterior reference axis used in that study. Regarding the apparent reduction in nasal valve CSA along the centerline, patient movement by flaring the nostrils or slight changes in segmentation threshold may account for the change with respect to this orientation. As a separate check ^47^, direct measurement of nasal valve area on a cross-sectional plane at a 90 degree tangent to the palate using anatomical landmarks showed in contrast an increase in nasal valve area in accordance with previous results.

#### Surface area, cavity volume, surface area volume ratio

**Figure 3:**
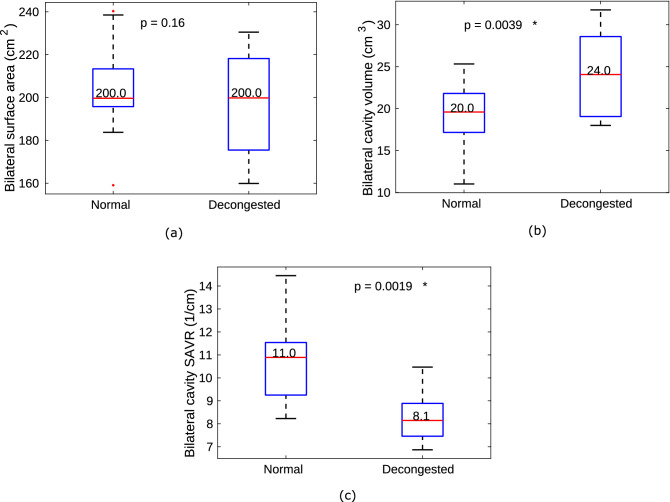
This figure shows effect of decongestion on nasal anatomy using Wilcoxon-signed rank test over the 10 subjects. On this and all similar data plots, the red line indicates the median value and the asterisk mark next to p value indicates statistical significance at the level of 5$\%$ on this and subsequent plots. (**a**) bilateral cavity surface area, (**b**) bilateral cavity volume, and (**c**) bilateral cavity surface area to cavity volume ratio (SAVR). Decongestant increases the cavity volume significantly, while the cavity surface area does not, leading to a significant SAVR change.

To quantify overall changes in geometry, cavity SA, CV, and the SAVR are compared before and after decongestion (Fig. 3).

Decongestion is found to significantly increase nasal cavity volume, but not cavity surface area. Consequently decongestion significantly reduces SAVR (median SAVR reducing from 11 to 8.1 $\hbox {cm}^{-1}$).

**Figure 4:**
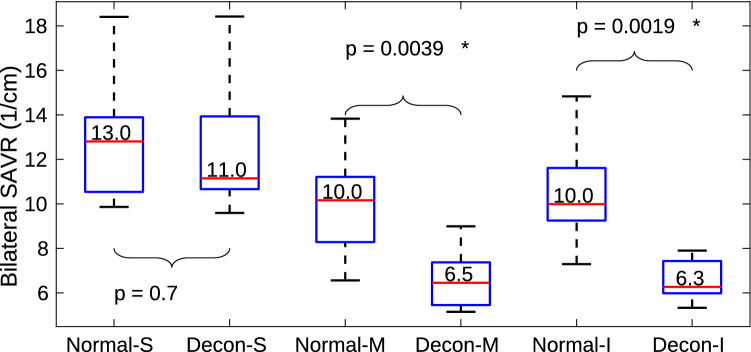
Nasal cavity surface area to volume ratio (SAVR) after decongestion in superior (S), middle (M) and inferior (I) parts of the cavity as defined in Fig.1 across the 10 subjects. The nasal cavity surface area volume ratio of the superior cavity does not change significantly after decongestion. However significant changes occurred in the middle and inferior regions.

The effect of decongestion was less marked in the superior parts of the nasal cavity compared with changes observed in middle and inferior regions. This is illustrated in the data ploted in Fig. 4, which compares SAVR in three vertical sections of the nasal cavities (defined in Fig. 1). SAVR in the superior cavity (S) reduces from 13 to 11 $\hbox {cm}^{-1}$ after decongestion, but the difference is not statistically significant. However, SAVR for the middle (M) and inferior (I) sections shows significant reduction with decongestion, with p = 0.004 for the middle and p = 0.002 for the inferior cavity. Figure 4 also shows that the superior section of the cavity has a larger median SAVR than the other two sections, in both normal and decongested conditions.

### Effect of decongestion on nasal airflow

#### Reynolds number

**Figure 5:**
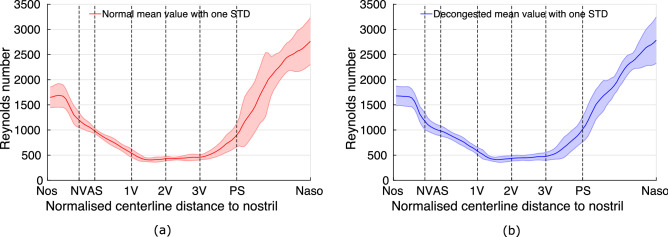
Comparison of the mean Reynolds number over 10 subjects throughout the nasal cavity before and after decongestion at flowrate of 30 L.min$^{-1}$. Left (**a**): the red curve represents the Reynolds number for normal state cavities. Right (**b**): blue curve represents the decongested cavities. The results show the mean Reynolds number remains relatively constant before and after decongestion. The shaded area represents the variations around mean value plus and minus one standard deviation.

Figure 5 shows the mean Reynolds number variation through the nasal passage from anterior to posterior. The Reynolds number is much higher in the anterior cavity (before 1V plane), and posterior cavity (after PS plane) compared to the middle section of the nasal cavity (between 1V and PS plane). The mean Reynolds number in the region of the cavity between AS and PS planes is below 1000 and less than 500 around plane 2V.

According to formula 5, for a specific flowrate, the Reynolds number only depends on the perimeter. Consequently the Reynolds number stays nearly unchanged which is mainly because the perimeter of the cavity remains largerly unaltered before and after decongestion (appendix D).

#### Nasal resistance

**Figure 6:**
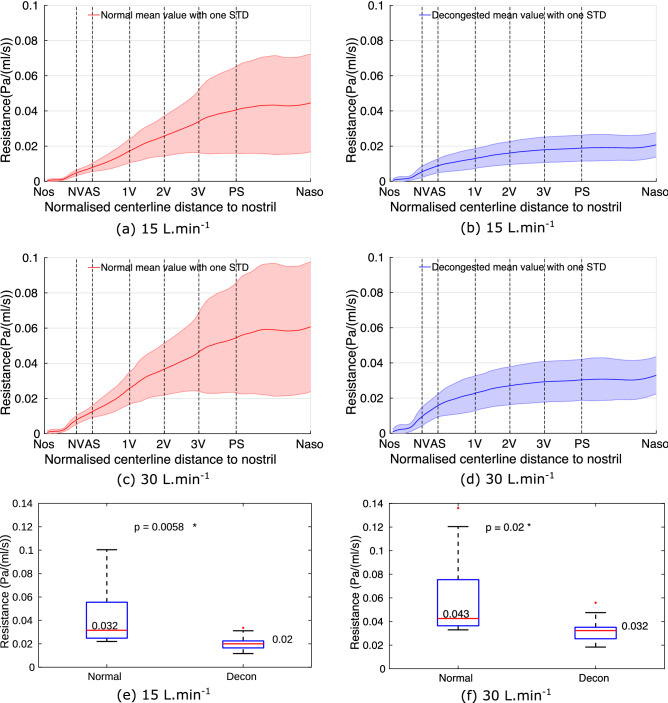
Figure (**a**–**d**) shows the cumulative resistance. (**a**,**b**) are the mean cavity resistance distribution of all 10 subjects along the cavity centerline before and after decongestion, respectively. (**c**,**d**) shows the same but for flowrate at 30 L.min$^{-1}$. The boxplots (**e**,**f**) show the effect of decongestant on the total nasal resistance at 15 L.min$^{-1}$ and 30 L.min$^{-1}$. The results show that decongestion can significantly reduce nasal resistance for both at 15 L.min$^{-1}$ and 30 L.min$^{-1}$.

Figure 6 shows the cumulative CSA-averaged intranasal resistance distribution for the normal and decongested cases at both 15 and 30 L.min$^{-1}$.

The increase in cumulative resistance (slope of the curve) is steeper in the anterior part (around the nasal valve, NV) of the cavity, excluding the nostril region for all the cases. This distribution of nasal resistance is also reflected by looking at the pressure scalar on one sagittal plane both before and after decongestion (as shown in Appendix C). Posterior to the AS plane, resistance increases at a nearly constant rate along the centerline until the PS plane, but at a lower rate than in the NV region. There is nearly no increase in resistance between PS and Naso planes of the cavity for both normal and decongested cavities. Furthermore, by comparing the total resistance before and after decongestion, the mean total resistance of decongested cavities is approximately half of that of the congested cavities. Lastly, comparing the respective standard deviations, there is higher inter-subject variability in nasal resistance of normal cavities than for the decongested cavities. After increasing the flowrate from 15 to 30 L.min$^{-1}$, the resistance distribution pattern remains nearly identical except the total resistance is approximately 1.4 times larger.

**Figure 7:**
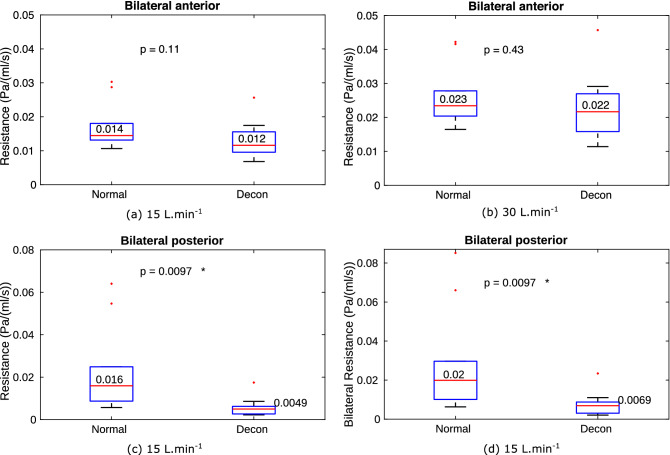
The anterior is enclosed by plane at nostril and plane 1V, the posterior is represented by plane 1V and the posterior septum plane. The results shows decongestion significantly reduces the resistance in the posterior part of the cavity at both flowrates ( (**c**) for 15 L.min$^{-1}$ and (**d**) 30 L.min$^{-1}$) at the level of 5$\%$, while the anterior cavity shows no significant effect, as shown between (**a**,**b**).

Overall the results show there is a significant decrease of resistance at both 15 and 30 L.min$^{-1}$ after decongestion, p=0.006 and 0.02, respectively. The reduction in overall resistance with decongestion can be ascribed purely to the drop in the posterior resistance, as shown in Fig. 7 at both flowrate.

#### Nasal resistance partitioning: inferior to superior

**Figure 8:**
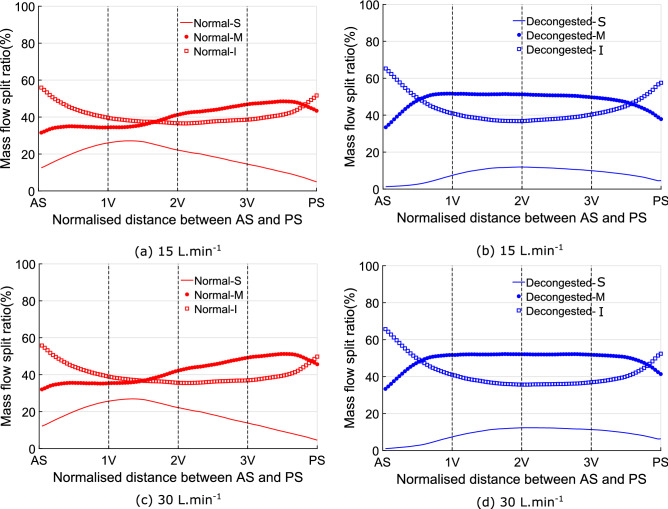
The effect of decongestant on the mean mass flow distribution between the superior, middle, and inferior partitions of the 10 nasal cavities. Plots (**a**,**b**) are the results for 15 L.min$^{-1}$ flowrate and plot (**c**,**d**) are the results for 30 L.min$^{-1}$.

Changes in the partitioning of flow between superior, middle and inferior regions of the nasal cavity are shown in Fig. 8.

The results show little dependence on flowrate. After decongestion, in the superior portion of the cavity, there is a marked reduction of the flow; flow through the middle cavity increases, while the proportion of mass flow through the inferior region remains nearly unchanged.

In the normal state, the ratio of mass flow into the middle of the nasal cavity starts to increase around the location of the head of middle turbinate (after 1V and before 2V plane). However, in the decongested cavity, the larger CSA after the nasal valve directs flow into the middle and inferior regions of the cavity immediately redirecting flow away from the superior cavity.

**Figure 9:**
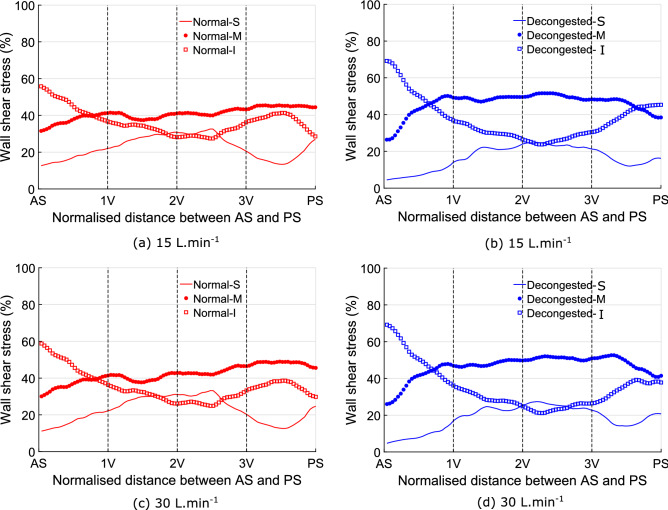
The effect of decongestion on the mean wall shear stress distribution over superior, middle, and inferior partitions of the 10 nasal cavities. Plot (**a**,**b**) are the results for 15 L.min$^{-1}$ flow rate and plot (**c**,**d**) are the results for 30 L.min$^{-1}$.

Comparing wall shear stress (WSS), Fig. 9 shows the proportional WSS loading to be higher in the inferior and middle regions of the nasal cavity than in the superior region, except around plane 2V, where the WSS loading of the superior region is similar or even higher than the inferior cavity. This pattern stays nearly unchanged after the application of decongestant.

From plane AS to PS, the WSS loading in the middle cavity exhibits a slight increase. However, the WSS loading in the inferior part of the cavity decreases then increases. The minimum point is located posterior to plane 2V. After decongestion, the WSS loading from plane AS to PS shows similar trends to those observed in the normal state, but with a larger variation in mean WSS loading between superior, middle and inferior parts. Decongested cavities show an abrupt increase of WSS loading in the middle cavity, after plane AS whereas only a slight increase of WSS loading is observed in the same part of the cavity in the normal states. Similar patterns can also be found near plane PS of normal and decongested cavities.

## Discussion

This study investigates the effect of decongestion on inspiratory airflow distribution in 10 healthy volunteers. Decongestion increases the average nasal CSA in the main turbinated region by approximately 1.4 times (Fig. 2). However, this change is minimized in the anterior nose (before AS plane). The anterior nose (around nasal valve) is made of various structures that have varying degrees of mobility: septal cartilage (rigid), inferior portion of the superior lateral cartilage (limited mobility), the erectile tissue of the nasal septum and inferior nasal turbinate head^48^. On the contrary, the turbinated region (between plane 1V and PS on Fig. 1) is surrounded by structures lined with erectile tissue which shrinks extensively due to the application of decongestant, leading to an increase of nasal cavity CSA. The reduction in the volume of the nasal turbinates due to decongestion causes a large increase in nasal cavity cross-sectional area posterior to the nasal valve (as shown in the Fig. 2 between AS and 1V plane). Further posterior at both normal and decongested states, the maximum CSA is located around the posterior septum where the two sides of the nasal cavity join.

The mean CSA of the normal nasal cavity matches measurements made previously by Zhao et al.^31^. The large offset of the cross-sectional area at the anterior part (before NV plane) of the cavity is caused by a different orientation of cross-sectional planes between this study and planes defined by Zhao et al.

SA of the nasal cavity is not changed significantly after decongestion, although the nasal CV increases (Fig. 3). As a result, the SAVR of the nasal cavity decreases significantly. In general, nasal cavities with higher SAVR are expected to exhibit larger resistance to airflow than cavities with lower SAVR, with the SAVR change effected by decongestion reducing median values from 0.032 to 0.02 Pa. s. ml$^{-1}$ at flowrate 15 L.min$^{-1}$, as shown in Fig. 6. On average, decongestion decreased nasal resistance by approximately 50% at both 15 L.min$^{-1}$ and 30 L.min$^{-1}$. In addition, increasing the flowrate was found only to change the magnitude of the airway resistance, but not the distribution of resistance along the cavity length.

Figure 7 showed that the majority of the change in nasal resistance due to decongestant occurs in the posterior nasal cavity (posterior to plane 1V defined in Fig. 1). In both normal and decongested geometries, resistance in the anterior cavity is dominated by the constriction around the nasal valve, which does not change significantly in CSA due to decongestant. However, CSA in the turbinates region (posterior to plane 1V) does expand due to decongestant, greatly reducing resistance in that region. For attached flow, WSS, which is determined by near wall velocity gradient, is the main source of resistance rather than high frequency eddies in the bulk flow. For a specific duct, mean flow velocity depends solely on flow rate so that zones of higher flow are expected to be associated with higher WSS. Consequently resistance in the superior, middle and inferior cavity is reflected by the ratio of WSS loading which in turn depends on the mass flow split ratio. The strength of this association is reflected by the similarity in the distributions for these quantities as shown in Figs. 8 and 9.

The Reynolds number of a flow is often used as a predictor of the presence of turbulence. However, in the nasal passages, the complex shape of the nasal cross-section means there is no obvious representative length. The hydraulic diameter (four times the cross-sectional area divided by its perimeter) of a cross-section is often used, although it may not always be the best choice, given that the perimeter of the nasal cavity includes narrow regions between the turbinates, while little airflow may pass through these regions. For a given flow rate, Reynolds number shows similar values before and after decongestion as explained above. This suggests the flow state should remain largely unaltered despite the change in geometry associated with decongestion, so that at comparable flow rates, similar flow models (laminar/turbulent) would be approporiate. Future studies could confirm this by measuring turbulent intensities in both cases.

The results indicate that at both flow rates, the superior cavity carries proportionally the lowest WSS both before and after decongestion, mainly due to the low mass flow to this region. More specifically, from anterior to posterior, the middle and inferior cavity carry approximately 40$\%$ of the total flow, while the superior cavity only accounts for roughly 20$\%$ before decongestion, whilst the middle cavity becomes both the dominant flow path and corresponding WSS load post decongestion. Similar results can be found at 15 and 30 L.min$^{-1}$. In addition, around 1V (after plane 1V for normal cavities while before plane 1V for decongested cavities), more flow goes into middle cavity (redirected by nasal turbinates), the proportion of WSS in the middle cavity gradually increases and eventually exceeds the inferior cavity. After decongestion, the proportion of WSS in the superior cavity reduces by nearly half on average, from approximately 20$\%$ to 10$\%$. The middle and inferior cavity WSS distributions are similar but the difference between them more pronounced in decongested compared to normal cavities. The change of flow rate has little effect on the WSS distribution for all partitions. This agrees with previous findings by Bates et al.^41^ who showed that the pattern of flow within the nose establishes at low flow rates and then does not change significantly, even if the magnitude of the flow changes.

While the results show a reduction in SAVR due to decongestant reduces nasal resistance, further work is necessary to assess whether this is beneficial. The high SAVR of the nose in the normal state facilitates the other functions of the nose such as heating and humidifying inhaled air, and olfaction. Additionally, previous work from Zhao et al.^49^ has shown that the sensation of nasal airflow depends on the cooling of nasal mucosa by inhaled air rather than resistance. Therefore a reduction in nasal resistance due to decongestant may not correlate with improved perception of airflow.

This study focused on subjects without nasal obstruction, unlike the study of Cherobin et al.^9^. Future studies should apply the same regional analysis to subjects with nasal obstruction to compare with the findings reported here for normal subjects, to determine whether the region of nasal obstruction responds to decongestant in the same way as healthy nasal anatomy, and to determine whether nasal resistance reduces in those subjects, or is limited by the obstruction. This study has provided a methodology to address those questions. This study demonstrates changes in nasal anatomy and airflow due to nasal decongestant applied via droplets. However, clinically it is also common to apply decongestant via nasal sprays which may distribute decongestant to different parts of the nasal cavity. Future studies may focus on the effect of different application modalities on changes to nasal anatomy and airflow.

The main limitation of this study was the small number of subjects (10) due to the time cost of segmentation and computational expense of CFD simulations. More sophisticated CFD models incorporating the temporal variations of respiration, and the effects of nasal heating and humidification as well as imaging at different times to capture the effects of the nasal cycle could be included in future studies.

## Conclusions

Firstly, decongestion was found to increase nasal cavity cross-sectional area (by 1.4 times on average), and significantly decreases SAVR from a median 11 to 8.1. Although decongestion significantly increases CSA, the cross-sectional perimeter is marginally altered. As a result, the local Reynolds number based on hydraulic diameter is hardly altered, suggesting similarity in local flow stability. Secondly, results indicate that decongestion not only reduced the nasal cavity resistance (approximately 50$\%$), but minimized the variability of nasal resistance across the cohort. This finding indicates that applying decongestant regularises nasal cavity flow. Thirdly, although decongestion exposes more of the superior nasal cavity, paradoxically less flow was found to reach this portion due to the concomitant reduction in airway resistance induced in the inferior part of the cavity.

Future studies of nasal airflow which are based on images of the nose at a single time point could use the results presented here to indicate the probable range over which factors like nasal resistance could vary. Moreover the anatomical variations presented here may indicate appropriate variations in model geometries to be applied in related studies such as those concerning upper airway aerosol transport.

## Supplementary Information

Supplementary Information 1.

## Data Availability

The datasets generated during and/or analysed during the current study are available from the corresponding author on reasonable request.
