# Supplementary material for: The effect of decongestion on nasal airway patency and airflow
Source: Sci Rep. 2021 Jul 13;11:14410. doi: 10.1038/s41598-021-93769-6 (PMC8277849; doi:10.1038/s41598-021-93769-6)
Supplement: Supplementary file 1 — Supplementary Information 1. [file 41598_2021_93769_MOESM1_ESM.pdf]

The Effect of Decongestion on Nasal Airway Patency and Airflow

Qiwei Xiao[1,2], Alister J. Bates[1,2,3], Raul Cetto[4], and Denis J. Doorly[4,*]

[1]Center for Pulmonary Imaging Research, Cincinnati Children's Hospital Medical Center, Cincinnati, Ohio, USA
[2]Division of Pulmonary Medicine, Cincinnati Children's Hospital Medical Center, Cincinnati, Ohio, USA
[3]Department of Pediatrics, University of Cincinnati, Cincinnati, Ohio, USA
[4]Department of Aeronautics, Imperial College London, South Kensington Campus, London, SW7 1AZ, United Kingdom
[*]d.doorly@imperial.ac.uk

ABSTRACT

Nasal decongestant reduces blood flow to the nasal turbinates, reducing tissue volume and increasing nasal airway patency. This study maps the changes in nasal anatomy and measures how these changes affect nasal resistance, flow partitioning between superior and inferior cavity, flow patterns and wall shear stress.

High-resolution MRI was applied to capture nasal anatomy in 10 healthy subjects before and after application of a topical decongestant. Computational fluid dynamics simulated nasal airflow at steady inspiratory flow rates of 15 L.min^{-1} and 30 L.min^{-1}.

The results show decongestion mainly increases the cross-sectional area in the turbinate region and SAVR is reduced (median approximately 40% reduction) in middle and lower parts of the cavity. Decongestion reduces nasal resistance by 50% on average, while in the posterior cavity, nasal resistance decreases by a median factor of approximately 3 after decongestion. We also find decongestant regularises nasal airflow and alters the partitioning of flow, significantly decreasing flow through the superior portions of the nasal cavity. By comparing nasal anatomies and airflow in their normal state with that when pharmacologically decongested, this study provides data for a broad range of anatomy and airflow conditions, which may help characterize the extent of nasal variability.

Appendices

A Mesh convergence

Figure below shows simulation mesh comparison between 4 million and 6 million setup over several pre-defined cross-sectional planes which indicates the visual difference.

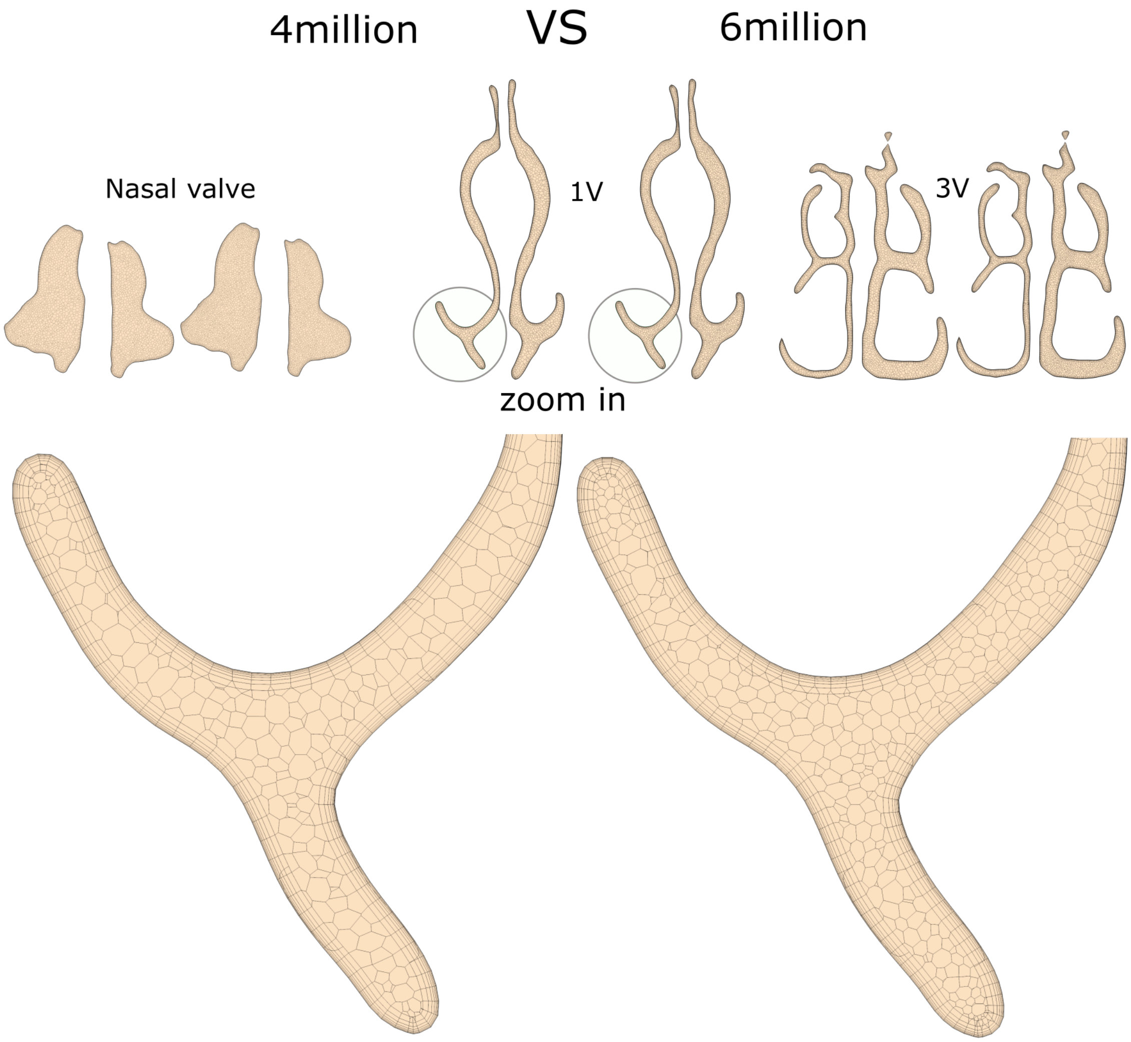

Figure 1. This figure shows the mesh details on three pre-defined planes: NV, 1V and 3V between simulation of 4 million elements and 6 million elements.

The quantitative results of the mesh convergence study is shown in the table below:

Mesh amount (millions)	1.9	2.6	3.0	4.0	5.2	6.5
Overall pressure loss (Pa)	23.2	23.8	24.1	24.1	24.5	24.6
Relative error (%)	6	3	2	2	0.4	0

Table 1. This table shows the total pressure loss along with the increase of total simulation mesh element.

B Geometric details of all the subjects

Table below shows the measurement of nasal anatomy of each subject.

Plane	Nos	NV	AS	1V	2V	3V	PS	Naso
Mean	0	0.12	0.18	0.32	0.45	0.58	0.72	1
STD	0	0.03	0.04	0.04	0.03	0.04	0.04	0
Mean(mm)	0	13.5	20.3	36.0	50.6	65.3	81.0	113
Subject-A Normal(mm)	0	15.0	22.5	40.1	56.3	72.6	90.1	125.2
Subject-A Decon(mm)	0	14.5	21.8	38.8	54.5	70.2	87.2	121.1
Subject-B Normal(mm)	0	12.9	19.4	34.5	48.5	62.5	77.6	107.8
Subject-B Decon(mm)	0	12.5	18.7	33.3	46.8	60.4	75.0	104.1
Subject-C Normal(mm)	0	14.4	21.6	38.4	54.0	69.5	86.3	119.9
Subject-C Decon(mm)	0	13.9	20.9	37.1	52.2	67.2	83.4	115.9
Subject-D Normal(mm)	0	15.9	23.9	42.5	59.8	77.1	95.7	132.9
Subject-D Decon(mm)	0	15.3	22.9	40.7	57.2	73.8	91.6	127.2
Subject-E Normal(mm)	0	14.2	21.3	38.0	53.4	68.8	85.4	118.6
Subject-E Decon(mm)	0	13.9	20.8	37.0	52.1	67.1	83.3	115.7
Subject-F Normal(mm)	0	15.1	22.6	40.2	56.5	72.8	90.4	125.6
Subject-F Decon(mm)	0	14.8	22.2	39.4	55.4	71.4	88.6	123.1
Subject-G Normal(mm)	0	14.9	22.3	39.6	55.8	71.9	89.2	123.9
Subject-G Decon(mm)	0	14.8	22.2	39.5	55.6	71.6	88.9	123.5
Subject-H Normal(mm)	0	12.5	18.8	33.4	47.0	60.6	75.2	104.5
Subject-H Decon(mm)	0	12.6	18.9	33.6	47.2	60.8	75.5	104.9
Subject-I Normal(mm)	0	13.3	19.9	35.4	49.8	64.1	79.6	110.6
Subject-I Decon(mm)	0	12.9	19.3	34.3	48.3	62.2	77.3	107.3
Subject-1 Normal(mm)	0	14.7	22.0	39.2	55.1	71.0	88.1	122.4
Subject-1 Decon(mm)	0	14.8	22.2	39.5	55.6	71.6	88.9	123.5

Table 2. This table shows the dimension of each nasal cavity in this study with mean and standard deviation values. All the positions of pre-defined planes also listed for easy access.

C Flow regimes on plane

This section shows flow pattern over a specific plane.

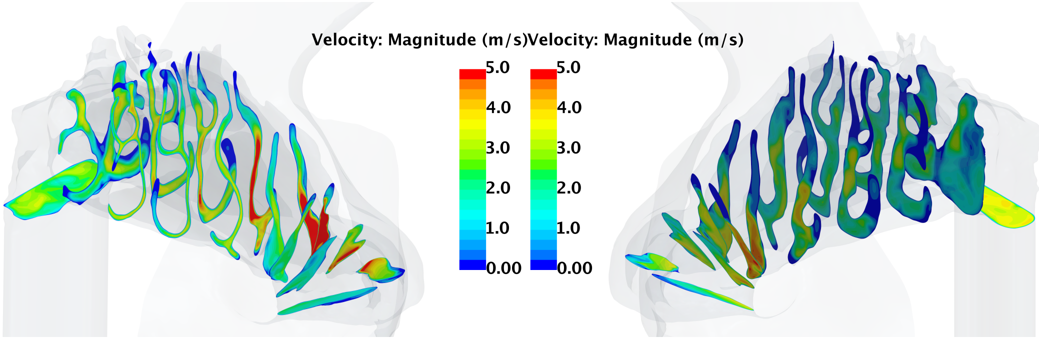

(a)

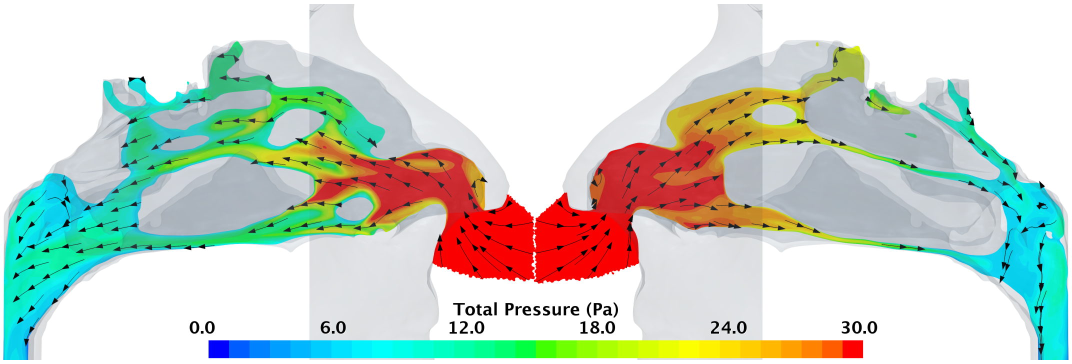

(b)

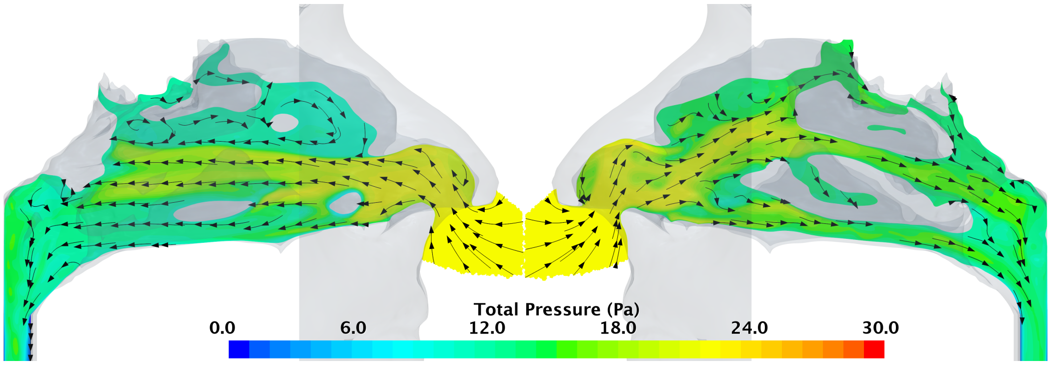

(c)

Figure 2. Sub-figure (a) shows the comparison of flow velocity on multiple pre-defined cross-sectional planes between before and after decongestion. Sub-figure (b) demonstrate total pressure contour on one sagittal plane, left side represents left cavity while right side represents right cavity of normal state cavity. Sub-figure (c) shows the same information as (b) but for cavity after decongestion.

D Mean perimeter variations before and after decongestion

Figure below shows the comparison of nasal cavity perimeter before and after decongestion. It can be seen that cavity perimeter remains nearly identical before and after decongestion.

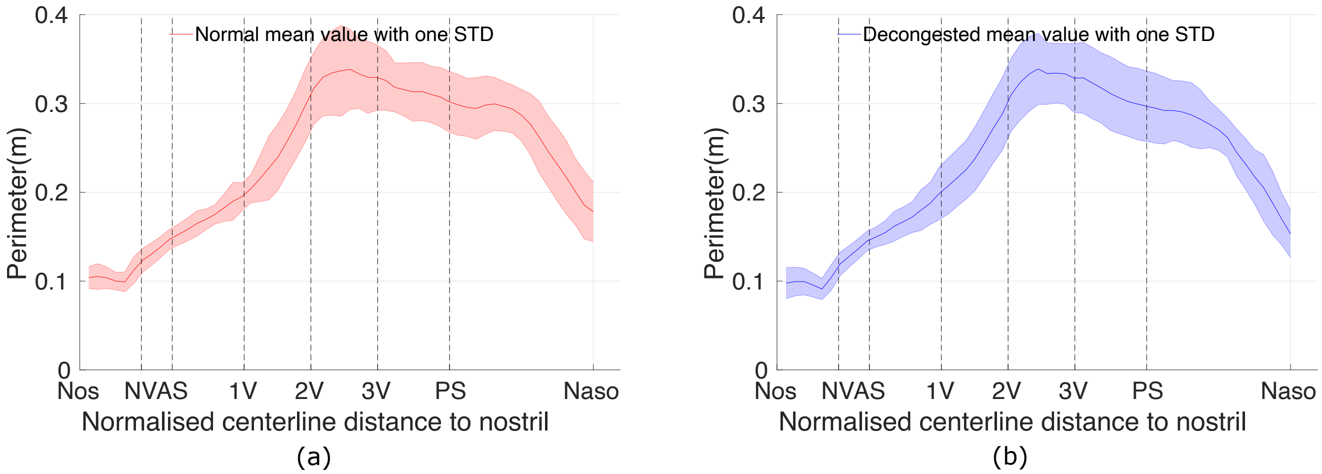

Figure 3. Sub-figure (a) shows the mean perimeter along with the cavity centerline. The shaded area represents one standard deviation. Similar result showed in sub-figure (b) of cavities after decongestion. This comparison indicates the variation of mean perimeter before and after decongestion remains nearly unchanged.

E Flow modelling comparison

Laminar, k-ω and large eddy simulation are conducted to explore the effect of the flow model on overall nasal pressure drop predictions and result shows the effect is minor and can be neglected.

Turbulence modelling	Laminar	k-ω	LES
Overall pressure loss (Pa)	24.1	23.6	24
Relative error (%)	0.4	1.6	0

Table 3. This table shows the total pressure loss between different flow model.
